# A mixed methods study of researchers’ experiences of developing core outcome sets

**DOI:** 10.1186/1745-6215-16-S2-P60

**Published:** 2015-11-16

**Authors:** Elizabeth Gargon, Bridget Young, Paula Williamson

**Affiliations:** 1 University of Liverpool, Liverpool, UK

## Background

A systematic review of core outcome sets (COS) identified 198 COS [1]. A range of methods were used to develop COS. Furthermore, 164/178 studies that described the methods used did not provide an explanation regarding their choice of methodology. There is little guidance about how to conduct or report COS studies and it is currently uncertain which of these methods are the most suitable, feasible and efficient. It is important to investigate COS developers’ choice of approach as this is a new area of research, and in order to formulate guidance in this area we need to try and understand the current situation, including the influences of methodological choices being made.

## Methods

We have used a mixed methods approach, using qualitative methods (semi-structured interviews) and a web-based survey.

## Results

Interviews are currently underway. The survey was sent out to 169 COS developers, with 81/169 responses. Methodological decisions were based most commonly on previous work, expert advice or own experience. Challenges of this work included resources (time, funding and technology), achieving consensus, a lack of data and challenges with involving patients in the process.

## Conclusion

In order to develop methodological guidance for COS development we need to try to understand what factors have informed the ways in which researchers have developed COS. This is the first insight into COS developers’ choice of methodology and their experiences of the process. These results will provide a more comprehensive account of COS development, ultimately facilitating the formulation of guidance in this area.
